# Complete Genome Sequence of *Geobacillus thermodenitrificans* T12, A Potential Host for Biotechnological Applications

**DOI:** 10.1007/s00284-017-1349-0

**Published:** 2017-09-12

**Authors:** Martinus J. A. Daas, Bastienne Vriesendorp, Antonius H. P. van de Weijer, John van der Oost, Richard van Kranenburg

**Affiliations:** 1 Laboratory of Microbiology, Wageningen University, Stippeneng 4, 6708 WE Wageningen, The Netherlands; 2 Corbion, Arkelsedijk 46, 4206 AC Gorinchem, The Netherlands

## Abstract

In attempt to obtain a thermophilic host for the conversion of lignocellulose derived substrates into lactic acid, *Geobacillus thermodenitrificans* T12 was isolated from a compost heap. It was selected from over 500 isolates as a genetically tractable hemicellulolytic lactic acid producer, requiring little nutrients. The strain is able to ferment glucose and xylose simultaneously and can produce lactic acid from xylan, making it a potential host for biotechnological applications. The genome of strain T12 consists of a 3.64 Mb chromosome and two plasmids of 59 and 56 kb. It has a total of 3.676 genes with an average genomic GC content of 48.7%. The T12 genome encodes a denitrification pathway, allowing for anaerobic respiration. The identity and localization of the responsible genes are similar to those of the denitrification pathways found in strain NG80-2. The hemicellulose utilization (HUS) locus was identified based on sequence homology against *G. stearothermophilus* T-6. It appeared that T12 has all the genes that are present in strain T-6 except for the arabinan degradation cluster. Instead, the HUS locus of strain T12 contains genes for both an inositol and a pectate degradation pathway. Strain T12 has complete pathways for the synthesis of purine and pyrimidine, all 20 amino acids and several vitamins except D-biotin. The host-defense systems present comprise a Type II and a Type III restriction-modification system, as well as a CRISPR-Cas Type II system. It is concluded that *G. thermodenitrificans* T12 is a potentially interesting candidate for industrial applications.

**Electronic supplementary material:**

The online version of this article (doi:10.1007/s00284-017-1349-0) contains supplementary material, which is available to authorized users.

## Introduction

Bioprospecting to discover novel organisms for the use in industrial applications is widely used and this strategy has led to an increased interest *Geobacillus spp.* [1, 3, 10, 16, 23, 25, 26, 31]. The *Geobacillus* genus, which was reclassified in 2001 from *Bacillus,* consists of Gram positive, rod shaped, spore-forming bacteria that thrive under thermophilic conditions and are capable of aerobic and anaerobic respiration [19]. Recently, Aliyu et al. have suggested that the genus *Geobacillus* consists of two distinct genera, *Geobacillus* and *Parageobacillus.* The latter genus includes *P. caldoxylosilyticus*, *P. thermoglucosidasius*, *P. antartcicus*, *P. toebii*, and *Parageobacillus* genomospecies 1 (NUB3621) [2]. The ability of *Geobacillus* spp. to metabolize C6 and C5 sugars, starchy substrates, and xylans is of particular interest for their application in biomass conversions [19, 31].

A range of recently reported applications of geobacilli, including the production of several (heterologous) proteins, fuels, and chemicals, underpin the potential of this genus [4, 5, 13, 23, 33, 36]. Still, a greater understanding of their genomes, metabolism, and the development of a robust genetic toolbox would facilitate the development of *Geobacillus* spp. into valuable whole-cell catalysts. Several strains of (*Para*)*Geobacillus* have proven to be genetically accessible, but the genomes of *Geobacillus stearothermophilus* NUB3621, *Parageobacillus thermoglucosidasius* DSM 2542, and *Geobacillus kaustophilus* HTA426 remain the only genetically accessible strains published to date [22, 31]. Although an impressive number of genome sequences have been completed in the past few years, the genomic information on geobacilli is still limited.

Here, we describe the complete genome sequence of *Geobacillus thermodenitrificans* T12, which is accessible in the GenBank database (CP020030-CP020032). Previously, Daas et al. [14] isolated *G. thermodenitrificans* strain T12 from compost and characterized this strain for its ability to co-ferment C5 and C6 sugars, as well as its ability to produce lactic acid and acetic acid from beechwood xylan. Furthermore, a relatively efficient transformation protocol has been developed for strain T12 [14]. In this paper, several genomic highlights are described, demonstrating the potential of strain T12 not only as an interesting whole-cell catalyst, but also as a valuable resource of useful genetic elements.

## Materials and Methods

### Media, Cultivation Methods, and DNA Isolation

*G. thermodenitrificans* T12 was isolated from a compost heap at Recom Ede B.V. in the Netherlands (52.043941 latitude° and 5.616682° longitude). It was demonstrated to co-ferment C6 and C5 sugars, to utilize xylan, and to be genetically accessible [14]. The isolated organism is a facultative anaerobic thermophile capable of denitrification [14]. It was grown in LB2 or MMy media at 65 °C in a rotary shaker with an agitation speed of 150 RPM. Genomic DNA of strain T12 was isolated from 10 ml of a logarithmic growing culture of an OD_600nm_ of 0.87 by using the MasterPure™ Gram Positive DNA Purification Kit (Epicentre, Madison, Wisconsin, USA) according to manufacturer’s protocol.

LB2 contains the following per liter: 10 g tryptone (Oxoid), 5 g yeast extract (Oxoid), 10 g NaCl and salts mix consisting of 0.25 g K_2_HPO_4_ (after autoclaving); 1 g NH_4_Cl; 3 g NaCl; 1.50 g Na_2_SO_4_; 0.08 g NaHCO_3_; 1 g KCl; 1.8 g MgCl_2_ × 6H_2_O; and 0.30 g CaCl_2_ × 2H_2_O; MOPS was added as a buffering agent and pH was set to 7.04 at room temperature. The medium was autoclaved for 20 min at 121 °C after which 1 ml filter sterile metal mix and 1 ml filter sterile vitamin solution were added. Metal mix contains the following per liter: 1.60 g MnCl_2_ × 6H_2_O; 0.1 g ZnSO_4_; 0.2 g H_3_BO_3_; 0.01 g CuSO_4_ × 5H_2_O; 0.01 g Na_2_MoO_4_ × 2H_2_0; 0.1 g CoCl_2_ × 6H_2_O; 0.7 g FeSO_4_ × 7H_2_O; 5 g CaCl_2_ × 2H_2_O and 20 g MgCl_2_ × 6H_2_O. Vitamin mix contains the following per liter: 0.1 g thiamine; 0.1 g riboflavin; 0.5 g nicotinic acid; 0.1 g pantothenic acid; 0.5 g pyridoxamine, HCl; 0.5 g pyridoxal, HCl; 0.1 g D-biotin; 0.1 g folic acid; 0.1 g *p*-aminobenzoic acid; 0.1 g cobalamin.

MMy medium contains the following per liter: 0.5 g yeast extract (Oxoid); 0.52 g K_2_HPO_4_; 0.23 g KH_2_PO_4_; 0.34 g NH_4_Cl; 8.37 g MOPS was used as a buffering agent and pH was set to 7.04 at room temperature. After autoclaving of the medium 0.14 g CaCl × 2H_2_O, 1 ml vitamin mix and 1 ml metal mix were added.

Glucose (10 g/l) was used as carbon source (unless stated otherwise) and separately autoclaved. Pectic substrates were used at a concentration of 5 g/l and were sterilized by dry incubation at 120 °C for 30 min. For plate cultures, 5 g/l gelrite (Carl Roth, Karlsruhe, Germany) was added. API 50 CHB/E test was inoculated from a 10 ml overnight LB2 culture of T12. Cells from the LB2 culture were centrifuged at 4000×g for 10 min and then transferred to the API 50 CHB/E Medium to obtain a cell density of 0.24 AUs at 600 nm. After inoculation, the API 50 CHB/E strips were inoculated for 48 h at 60 °C.

### Genome Sequencing, Assembly, and Annotation

The *G. thermodenitrificans* T12 genome was sequenced, assembled, and annotated by Baseclear B.V. (Leiden, The Netherlands). Pair-end sequence reads were generated by using the Illumina HiSeq2500 system followed by PacBio sequencing using the PacBio RS system. The quality of the Illumina FASTQ sequences was enhanced by trimming off low-quality bases with the “Trim sequences” option of the CLC Genomics Workbench version 7.0.4. Subsequently, the quality-filtered sequence reads were puzzled into a number of contig sequences. The data collected from the PacBio RS instrument were processed and filtered with the SMRT Analysis software suite. Filtering of the continuous long read (CLR) data was done by Read-length (>50), Subread-length (>50), and Read quality (>0.75). The average read length of the paired-end reads was 254.15 bp and those of PacBio were 2795 bp. Analysis of methylation patterns was included in the SMRT portal (v2.3), using the RS_Modification_and_Motif_Analysis.1 workflow. A summary of the discovered motifs is given in Table 1.

**Table 1:** Methylation analysis summary

MotifString^a^	CenterPos^b^	R-M type^c^	Modification type	Fraction^e^
5′-GATC-3′	1	III	m6A^d^	0.992
5′-TAAYNNNNNNRTTA-3′	2	I	m6A	0.983
5′-GCCAT-3′	3	II	m6A	0.957

^a^Detected motif sequence for this site such as “GATC”

^b^Position in motif of modification (0-based)

^c^Type of restriction modification system [35]

^d^N6-Methyladenosine (m6A) refers to a type of methyltransferase that prefers to methylate the adenosine base at the nitrogen-6 position

^e^The percent of time this motif is detected as modified in the genome

The assembly has been performed with the “De novo assembly” option of the CLC Genomics Workbench version 7.0.4. and the optimal k-mer size was automatically determined with KmerGenie [11]. The assembled contigs were linked and placed into super-scaffolds based on the alignment of the PacBio CLR reads. This alignment was performed with BLASR [9]. From the alignment the orientation, order and distance between the contigs were estimated by using the SSPACE-LongRead scaffolder version 1.0 [6]. The gapped regions within the super-scaffolds were closed in an automated manner by GapFiller version 1.10 [7]. Closing the gaps resulted in seven scaffolds: one that covers the T12 genome, two that cover the plasmids, and three contaminant scaffolds that were assembled and then removed from the dataset.

Genome annotation was performed on the assembled scaffolds by using the BaseClear annotation pipeline, which is based on the Prokka Prokaryotic Genome Annotation System (http://vicbioinformatics.com/). Genes were predicted with Prodigal V2 and translated CDSs were used to search the Uniprot database to identify EC number, CAZY number and function annotation. A domain analysis was performed with *hmmer*-*3* and signal peptides, with their corresponding cellular localization, were predicted by using *signalp v4.0*. To predict rRNAs and tRNAs, we used barrnap v0.2 and Aragorn v1.2.36, respectively.

The genome of *G. thermodenitrificans* T12 encompasses a 3.64 Mb chromosome and two plasmids of 59 and 56 kb that together contain 3676 genes and an average GC content of 48.71% (Table 2). The majority of genes predicted were assigned with a known function (2088 genes) and a total of 47 genes with a CAZy number were annotated (Table 3). The genome is available in the GenBank database (CP020030-CP020032).

**Table 2:** Genome statistics

Attribute	Value
Chromosome size (bp)	3,640,708
Plasmid 1 size (bp)	58,808
Plasmid 2 size (bp)	56,976
GC percentage chromosome	49.10
GC percentage plasmid 1	38.91
GC percentage plasmid 2	39.40
Number of genes	3676
Number of tRNA genes	94
Number of rRNA genes	30
Gram	+
Organism	*G. thermodenitrificans*

**Table 3:** Statistics of coding sequences (CDS)

Attribute	Value
Number of genes	3676
With a known function	2088
With a GO annotation	2379
With a CaZy number	47
With a signal peptide	146
Gene density (gene/Mbp)	977
Total gene size	3,233,578
Max gene size	7155
Min gene size	71
Average gene size	879

## Results and Discussion

**Fig. 1:**
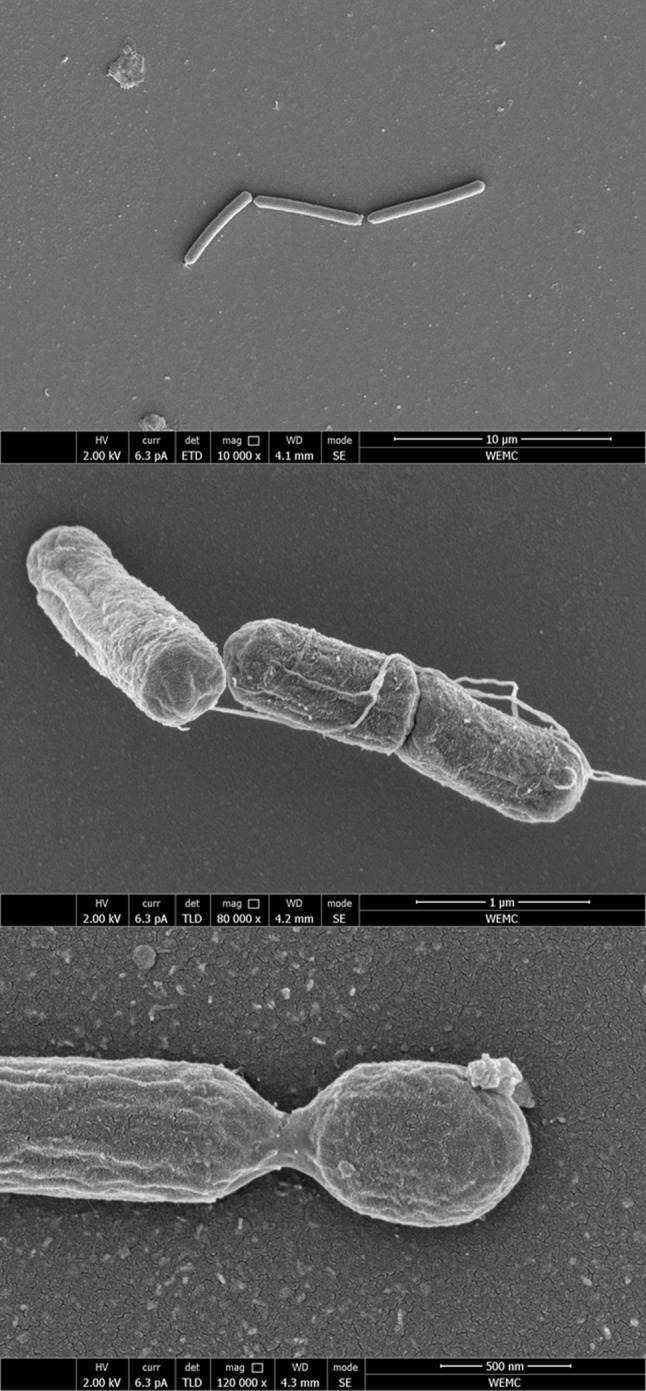
Scanning electron micrographs of *G. thermodenitrificans* T12

*G. thermodenitrificans* T12 is a rod-shaped, thermophilic, facultative anaerobic, Gram-positive bacterium that was isolated from compost (Fig. 1). The phylogeny of strain T12 was determined using its 16S RNA encoding gene. This sequence was used to create a phylogenetic tree that demonstrates its relationship to several *Geobacillus* type strains [14]. *G. thermodenitrificans* T12 was selected for whole genome sequencing based on its fermentation product profile, co-utilization of glucose and xylose, its ability to degrade xylan, and its amenability to transformation [14].

### Insights to the Genome

#### General Metabolism

Strain T12 is capable of utilizing a wide variety of carbohydrates (SI; Table S1) as was proven by fermentation test with an API 50 CHB/E test kit (BioMérieux, Marcy l’Etoile, France). We also determined the presence of complete pathways for the synthesis of purine and pyrimidine, all 20 amino acids and several vitamins except D-biotin. Pathways for the synthesis of these compounds are identical to those identified for *G. thermodenitrificans* NG80-2 (KEGG entry: T00496). The D-biotin pathway in strain T12 lacks the genes required for the conversion of Pimeloyl-(acp) to Dethiobiotin, however, a putative biotin transporter encoding gene (GTHT12_02457) was found.

#### Denitrification

*G. thermodenitrificans* T12 is capable of anaerobic respiration, reducing nitrate to molecular nitrogen [14]. The metabolic pathways of strain T12 involved in this reduction have been identified based on the annotated genome of *Geobacillus* sp. NG80-2, which was shown to be capable of denitrification. The genes encoding all enzymes of the denitrification pathway have been identified in its genome [18]. Two copies of the nitrate reductase operon *narGHJI*, responsible for the reduction of nitrate to N_2_O, are present in the genomes of both strain T12 and strain NG80-2. The homology of genes involved in both copies of the *narGHJI* operon is 99 to 100% sequence identity between the two strains. Not only does the denitrification cluster of T12 show high identity to their homologs of strain NG80-2, also their genomic localization is identical to the genomic localization in strain NG80-2.

The *nos* gene cluster in strain NG80-2 is required for the reduction of N_2_O to N_2_, but this cluster was not described for gram-positive bacteria until its discovery in strain NG80-2 [18]. We here reported the identification of genes encoding all enzymes for denitrification in strain T12 with 97 to 100% sequence identity to their orthologs of strain NG80-2.

#### HUS Locus

The hemicellulose utilization (HUS) locus of strain T12 contains 95 genes with a total size of 121 kb and is thereby the largest hemicellulolytic cluster reported for *Geobacillus* spp. [15]. The HUS locus shows extensive variability among geobacilli, even among strains of the same species [15]. This variability might be induced by the action of mobile genetic elements and the selection for desired traits based on the environment from which *Geobacillus* spp. are isolated.

When the HUS locus of strain T12 is compared to the HUS locus of *G. stearothermophilus* T-6, which is the most extensively studied HUS locus among *Geobacillus* spp., all T-6 genes are present in the T12 cluster, except for the arabinan degradation cluster. The arabinose metabolic genes of strain T12 show highest similarity to *Geobacillus* sp. 1MC16 with both strains lacking the gene cluster containing the extracellular and intracellular arabinase, although both do contain the enzymes needed for arabinose and arabinooligosaccharide degradation [8].

**Fig. 2:**
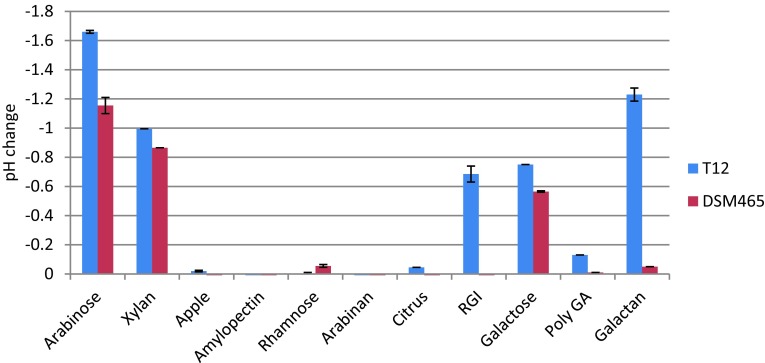
Acidification of MMy medium due to fermentation of various pectic substrates by *G. thermodenitrificans* strains T12 and DSM 465. Cultures were incubated at 65 °C for 24 h in a rotary shaker at 150 RPM. Xylan: beechwood xylan, Apple: apple pectin; Citrus: citrus pectin, RGI: rhamnogalacturonan I, Poly GA: poly galactic acid

Compared to T-6, the HUS locus of strain T12 has an additional inositol pathway, an oligopeptide-transport gene cluster, and a pectate degradation pathway. The inositol gene cluster (*iolG/DEBCA*) of strain T12 has a 93–100% amino acid identity to the inositol clusters of *G. thermodenitrificans* strains NG80-2, DSM 465, and G1MC16 [8]. Directly upstream of the inositol cluster in strain T12, an oligopeptide-transport gene cluster (*dppABCDFE*) is found which is not present in any other sequenced *G. thermodenitrificans* strain. This peptide-transport gene cluster is identical to its orthologs found in multiple other *Geobacillus* spp. [8]. *G. thermodenitrificans* strains T12, NG80-2, DSM 465, and G1MC16 also encode an α-mannosidase at the 5′-end of their HUS-locus. Surprisingly, a 23.3 Kb insert was found in between the mannosidase-encoding gene and the polypeptide transport gene cluster in strain T12. The insert contains a pectate degradation gene cluster containing both a pectate lyase (PL1) and a rhamnogalacturonyl hydrolase (GH88). Furthermore, genes GTHT12_1416 to GTHT12_1424 show high sequence identity to the rhamnogalacturonan degradation pathway of *Bacillus* spp. (SI; Table S2). When grown on various pectic substrates, strain T12 is capable of growth and organic acid production on both rhamnogalacturonan I and galactan. In contrast, strain *G. thermodenitrificans* DSM 465 cannot grow by converting these substrates and lacks the pectate degradation gene cluster in its HUS locus (Fig. 2). We conclude that the ability of strain T12 to ferment both rhamnogalacturonan and galactan most likely is the result of the pectate degradation cluster in its HUS locus.

### Additional CAZymes

Next to the carbohydrate active enzymes (CAZymes) present on the HUS locus, several additional genes have been localized that encode for CAZymes. Most notable are a starch degrading cluster (GTHT_00158 through GTHT_00163) encoding for both an intracellular α-amylase and an extracellular α-amylase, interspaced by a three component ABC transporter. In addition, two oligo-1,6-glucosidases were identified, located far apart on the genome. These oligo-1,6-glucosidases are predicted to degrade the oligosaccharides obtained from the degradation of starch by alpha-amylases, thereby complementing the starch degradation pathway. Besides the starch degradation pathway, we identified a 6-phospho-β-glucosidase (GTHT_01331) just upstream of a putative cellobiose-specific phosphotransferase uptake mechanism (GTHT_01332 through GTHT_01334). Additionally, three more β-glucosidases were identified scattered over the genome (GTHT_01847, GTHT_02694, and GTHT_2696). Two proteins annotated as peptidases (GTHT_02208 and GTHT_02311) showed high identity to proteins (EPR27003.1 and EPR26354.1, respectively) that were characterized as endo-glucanases [27]. However, although strain T12 demonstrated growth on carboxymethylcellulose, we were unable to detect degradation of this substrate (data not shown). Furthermore, on plasmid pGeo12b, a levanase encoding gene has been identified (GTHT_3754) located in between a three-component ABC transporter and a fructokinase, suggesting the ability of *G. thermodenitrificans* T12 to degrade fructans.

#### Host Defense Systems

On the genome of T12, multiple methylation motifs were discovered and the corresponding restriction-modification (R/M) systems were identified using the REBASE database (Table 1) [28]. A Type II R/M system was identified on plasmid pGeo12b, encoded by ORFs GTHT12_03786 through GTHT12_03788. Methylation pattern analysis on the T12 genome revealed 5′-GATC-3′ as potential Type II R/M system recognition sequence (Table 1). ORFs GTHT12_00809 and GTHT12_00810 encode a Type III R/M system that may recognize the 5′-GCCAT-3′ sequence retrieved from the methylation analysis. This Type III system is most closely related (99% amino acid identity) to a system in *Geobacillus* sp. PA-3. PacBio data also showed methylation at motif 5′-TAAYNNNNNNRTTA-3′, which is a typical Type I R/M system recognition sequence. Although we did find a putative Type I methylase (GTHT_3783), we were unable to discover the corresponding R and S subunits. The mentioned R/M systems might influence the genetic accessibility and most likely play a crucial role in phage resistance in the microbiome of the compost from which the T12 strain was isolated.

**Fig. 3:**
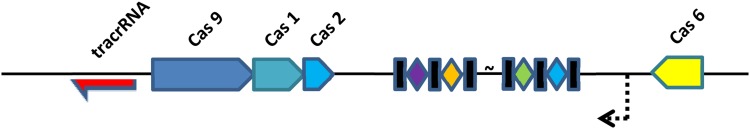
CRISPR-Cas Type II-C system architecture of *G. thermodenitrificans* T12. *Rectangles*: repeats; *diamonds*: spacers; *dashed arrow*: predicted promoter

In addition to the R/M systems, we discovered a Type II-C CRISPR-Cas system (called *Gt*Cas9 hereafter) on the genome of strain T12 (GTHT12_03309 through GTHT12_03401) [34]. The CRISPR-Cas locus architecture is a typical CRISPR-Cas Type II-C system [12], but contains a *cas6* gene located after the CRISPR array (Fig. 3). This *cas6* gene is likely to be a remnant of a Type I or Type III CRISPR-Cas system and is believed not to be part of the Cas9 locus in strain T12. The CRISPR-finder tool (http://crispr.i2bc.paris-saclay.fr/Server/) was used to identify the CRISPR array that contains 11 repeat sequences (36 bp) interspaced by ten spacer sequences (29–31 bp). The tracrRNA sequence was predicted by searching for sequences with strong complementarity to the repeat sequences in a 1-kb window flanking the CRISPR locus. A 34 bp tracr sequence (TCATAGTAACCCTGAGATCATTGCTGTGGTATAA) was found 164 bp upstream of the *cas9* gene. The tracrRNA, which can pair with the repeat sequence of the crRNA, is essential to crRNA maturation in this system [20, 21]. Spacers were blasted against all available databases of CRISPRTarget (http://bioanalysis.otago.ac.nz/CRISPRTarget/crispr_analysis.html) and were found to have hits against *Anoxybacillus flavithermus* WK1 phage DNA and several spacers matched against *Geobacillus* Virus E2 DNA. Virus E2 is present as prophage in the genomes of *G. thermodenitrificans* NG80-2 and *P. thermoglucosidasius* C56-YS93 but was not found in the genome of *G. thermodenitrificans* T12.

**Fig. 4:**
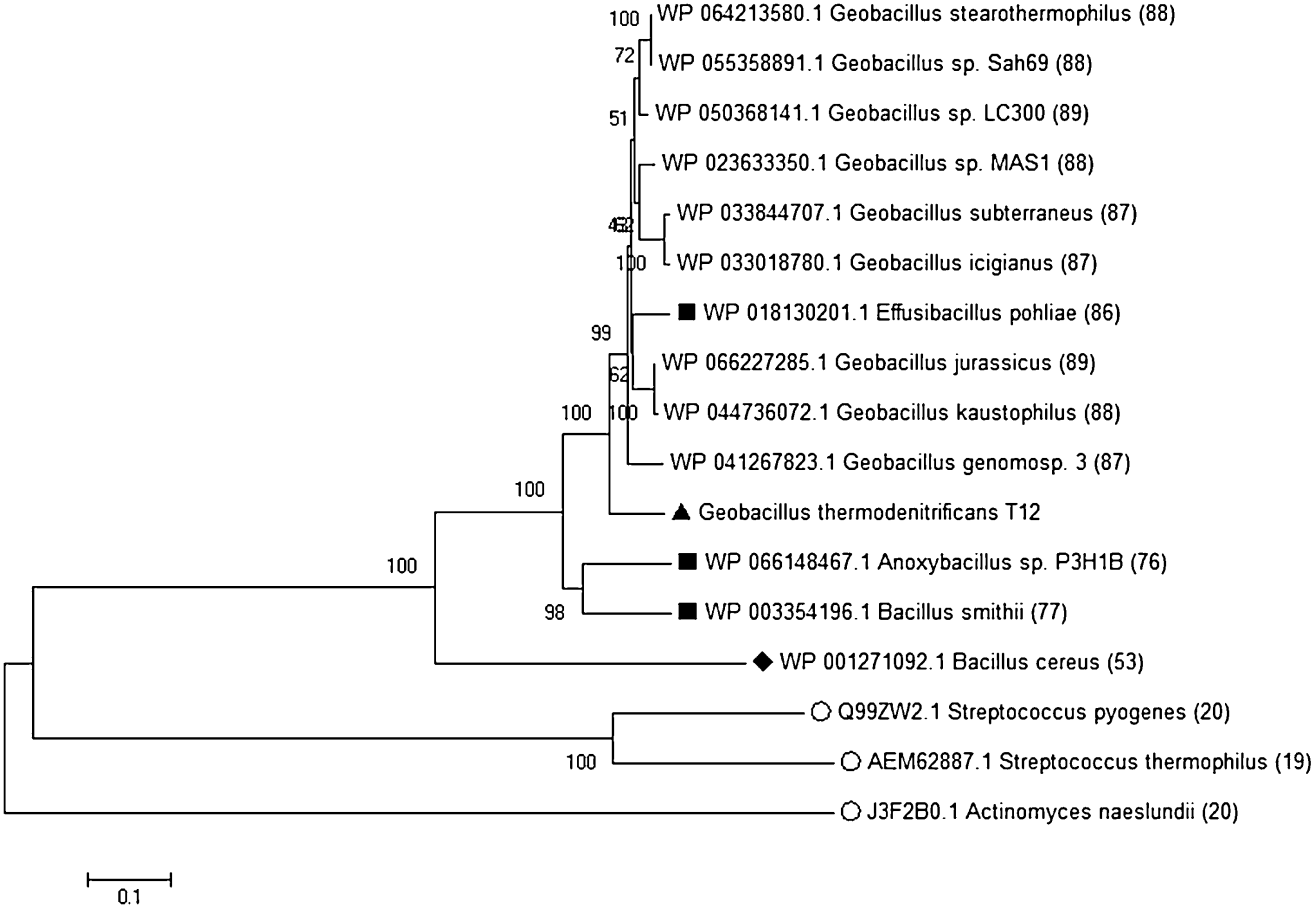
Neighbor-Joining tree of Cas9 protein sequences. The evolutionary history was inferred using the Neighbor-Joining method [29]. The percentage of replicate trees in which the associated taxa clustered together in the bootstrap test (1000 replicates) are shown next to the branches [17]. The tree is drawn to scale, with branch lengths in the same units as those of the evolutionary distances used to infer the phylogenetic tree. Evolutionary analyses were conducted in MEGA6 [32]. All sequences found in *Geobacillus* spp. were included, as well as currently well-characterized sequences (*Open circles*: *S. pyogenes, S. thermophiles, and A. naeslundii*), as well as the closest non-thermophilic species *Bacillus cereus* (*closed diamond*). Non-*Geobacillus* strains capable of thermophilic growth have been included (*closed squares*). For all sequences, the percentage of amino acid sequence identity to T12 is indicated after the strain name between *brackets*

The classification and evolution of Type-II CRISPR-Cas systems have previously been described and, in particular, three thermo-tolerant species have been identified which exhibit these Type-II systems. Still, these species grow optimally between 50 and 60 °C and not at 65 °C, as *G. thermodenitrificans* T12. To date there is no experimental evidence for active Cas9 proteins in thermophiles. Based on a comparative genome screening on the presence of Cas9 in bacteria, it was found that the Type II-C CRISPR-Cas system is only present in approximately 3.3% of all bacterial genomes [12]. However, comparative analysis to the non-redundant protein database of the NCBI revealed that Cas9 proteins are widespread among *Geobacillus* spp. A phylogenetic tree reveals the close relatedness of Cas9 proteins among thermophiles and their distinct mesophilic orthologs (Fig. 4). Although mesophilic Cas9 proteins show little sequence identity to *Gt*Cas9, protein sequence alignment against the well-characterized Cas9 proteins of *A. naeslundii* (Type II-C), *S. pyogenes*, and *S. thermophilus* (Type II-A) reveals the conservation of important active site residues in *Gt*Cas9 (Figure S1). CRISPR-Cas is often used for genome editing and the engineering toolbox expands rapidly [24, 30]. Functional analysis of *Gt*Cas9 for genome editing applications is ongoing (Mougiakos et al. unpublished results).

## Electronic supplementary material

Below is the link to the electronic supplementary material.

Supplementary material 1 (DOCX 849 kb)

Supplementary material 2 (DOCX 16 kb)

Supplementary material 3 (DOCX 16 kb)

Supplementary material 4 (DOCX 17 kb)
