# Supplementary material for: Complete Genome Sequence of Geobacillus thermodenitrificans T12, A Potential Host for Biotechnological Applications
Source: Curr Microbiol. 2017 Sep 12;75(1):49–56. doi: 10.1007/s00284-017-1349-0 (PMC5765199; doi:10.1007/s00284-017-1349-0)
Supplement: Supplementary file 1 — Supplementary material 1 (DOCX 849 kb) [file 284_2017_1349_MOESM1_ESM.docx]

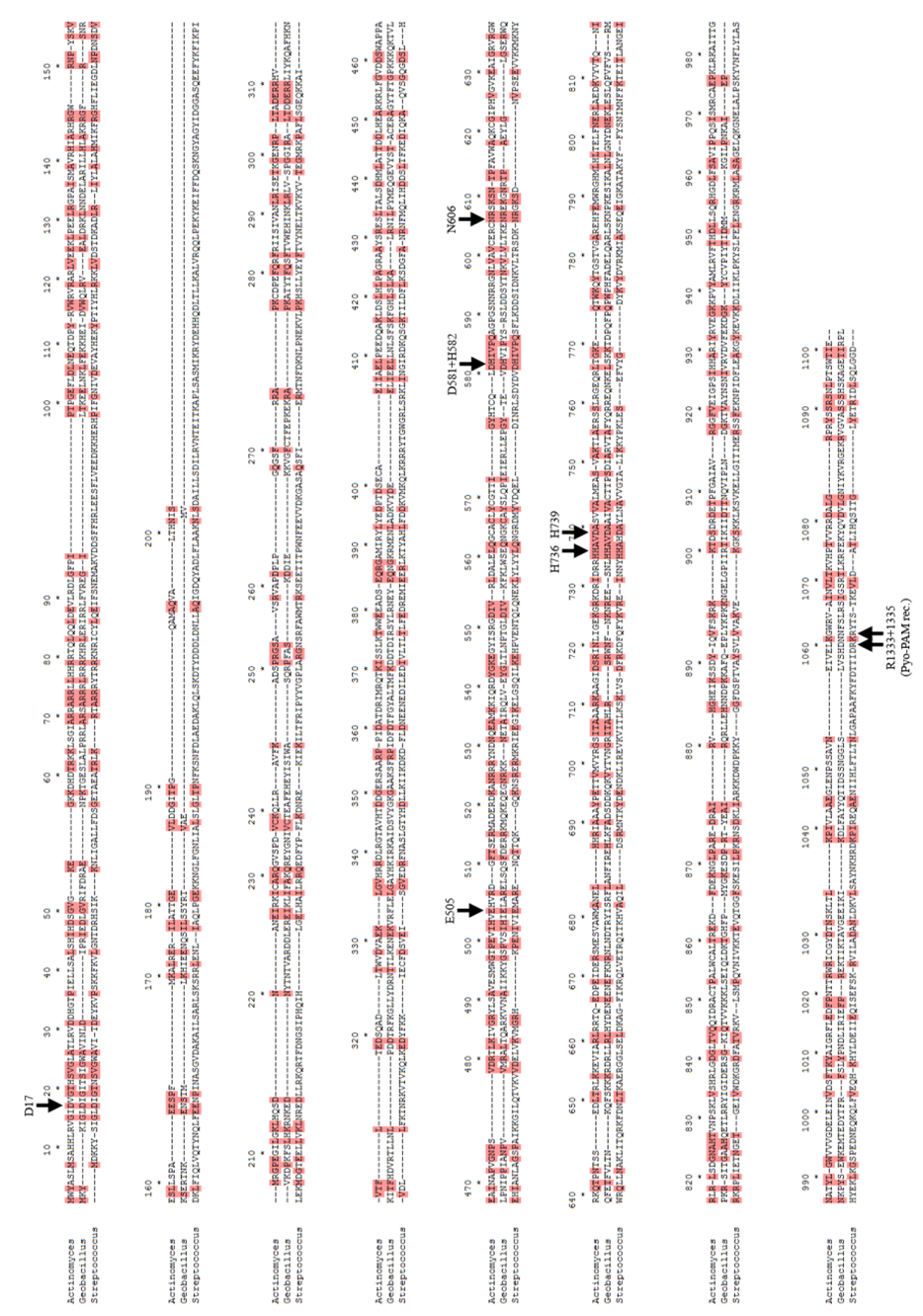


**Fig S1** Protein sequence alignment for *Gt*Cas9 (Type II-C) with well-characterized Type II-C (*A. naeslundii*/‘ana’) and Type II-A (*S. pyogenes*/’pyo’ and *S.* *thermophilus*) Cas9 sequences. Important active site residues are well conserved and indicated with black arrows. Protein domains as described for Ana-Cas9 and Pyo-Cas9 [40] are indicated with shaded boxes and similarly coloured letters. The PAM recognition domain has been determined for the *S. pyogenes* Type II-A system, but not for any Type II-C system and is therefore only indicated in the *S. pyogenes* sequence
