# Supplementary material for: Complete Genome Sequence of Geobacillus thermodenitrificans T12, A Potential Host for Biotechnological Applications
Source: Curr Microbiol. 2017 Sep 12;75(1):49–56. doi: 10.1007/s00284-017-1349-0 (PMC5765199; doi:10.1007/s00284-017-1349-0)
Supplement: Supplementary file 2 — Supplementary material 2 (DOCX 16 kb) [file 284_2017_1349_MOESM2_ESM.docx]

Table S1 Fermentation test results after 48h using API 50 CHB/E. Red: no acidification, orange: slight acidification, green: acidification

| 0 | Control |  | 25 | Esculin ferric citrate |  |
| --- | --- | --- | --- | --- | --- |
| 1 | Glycerol |  | 26 | Salicin |  |
| 2 | Eryhritol |  | 27 | D-Cellobiose |  |
| 3 | D-Arabinose |  | 28 | D-Maltose |  |
| 4 | L-Arabinose |  | 29 | D-Lactose (bovine origin) |  |
| 5 | D-Ribose |  | 30 | D-Melibiose |  |
| 6 | D-Xylose |  | 31 | D-Saccharose (sucrose) |  |
| 7 | L-Xylose |  | 32 | D-Trehalose |  |
| 8 | D-Adonitol |  | 33 | Inulin |  |
| 9 | Methyl-β-D-Xylopyranoside |  | 34 | D-Melezitose |  |
| 10 | D-Galactose |  | 35 | D-Raffinose |  |
| 11 | D-Glucose |  | 36 | Amidon (starch) |  |
| 12 | D-Fructose |  | 37 | Glycogen |  |
| 13 | D-Mannose |  | 38 | Xylitol |  |
| 14 | L-Sorbose |  | 39 | Gentiobiose |  |
| 15 | L-Rhamnose |  | 40 | D-Turanose |  |
| 16 | Dulcitol |  | 41 | D-Lyxose |  |
| 17 | Inositol |  | 42 | D-Tagatose |  |
| 18 | D-Mannitol |  | 43 | D-Fucose |  |
| 19 | D-Sorbitol |  | 44 | L-Fucose |  |
| 20 | Methyl-α-D-Mannopyranoside |  | 45 | D-Arabitol |  |
| 21 | Methyl-α-D-Glucopyranoside |  | 46 | L-Arabitol |  |
| 22 | N-Acetylglucosamine |  | 47 | Potassium gluconate |  |
| 23 | Amygdalin |  | 48 | Potassium 2-ketogluconate |  |
| 24 | Arutin |  | 49 | Potassium 5-ketogluconate |  |
