# Supplementary material for: Complete Genome Sequence of Geobacillus thermodenitrificans T12, A Potential Host for Biotechnological Applications
Source: Curr Microbiol. 2017 Sep 12;75(1):49–56. doi: 10.1007/s00284-017-1349-0 (PMC5765199; doi:10.1007/s00284-017-1349-0)
Supplement: Supplementary file 3 — Supplementary material 3 (DOCX 16 kb) [file 284_2017_1349_MOESM3_ESM.docx]

Table S2 The pectate degradation cluster of *Geobacillus thermodenitrificans* T12

| **Locus tag** | **Gene name** | **Product size (aa)** | **Subcellular Localization** | **Predicted protein function** | **Closest non-*Geobacillus* ortholog** | **Strain** | **AA identity (%)** | **Bitscore** | **E-value** |
| --- | --- | --- | --- | --- | --- | --- | --- | --- | --- |
| GTHT12_01416 | *kduI1* | 277 | - | 4-deoxy-L-threo-5-hexosulose-uronate ketol-isomerase 1 | *Bacillus* sp. | LF1 | 79 | 472 | 5.00E-167 |
| GTHT12_01417 | *kduD* | 256 | - | 2-dehydro-3-deoxy-D-gluconate 5-dehydrogenase | *Bacillus* sp. | JCM 19034 | 74 | 395 | 2.00E-137 |
| GTHT12_01418 | *GlpR* | 254 | - | Putative transcriptional repressor | *Bacillus* sp. | SA1-12 | 62 | 308 | 5.00E-103 |
| GTHT12_01419 | - | 404 | - | putative response regulatory protein | *Bacillus novalis* | NBRC 102450 | 41 | 286 | 2.00E-90 |
| GTHT12_01420 | *ypdA_1* | 599 | - | Sensor histidine kinase | *Bacillus novalis* | NBRC 102450 | 54 | 651 | 0.00E+00 |
| GTHT12_01421 | *pel* | 442 | Extracellular | Pectate lyase | *Bacillus* sp. | TS-47 | 100 | 916 | 0.00E+00 |
| GTHT12_01422 | *yesR* | 377 | - | Unsaturated rhamnogalacturonyl hydrolase YesR | *Bacillus novalis* | NBRC 102450 | 65 | 527 | 0.00E+00 |
| GTHT12_01423 | - | 449 | Cytoplasmic Membrane | ABC-type transport systems, binding protein | *Bacillus novalis* | NBRC 102450 | 67 | 654 | 0.00E+00 |
| GTHT12_01424 | *ugpE* | 279 | - | ABC transporter protein | *Bacillus* sp. | UNC41MFS5 | 77 | 459 | 5.00E-162 |
| GTHT12_01425 | - | 315 | - | ABC-type transport systems, permease protein | *Pelosinus fermentans* | JBW45 | 69 | 468 | 4.00E-164 |
| GTHT12_1502 | *yesY* | 273 |  | putative rhamnogalacturonan acetylesterase YesY | *Anoxybacillus geothermalis* | GSSed3 | 83 | 435 | 6.00E-153 |
