# Supplementary material for: Complete Genome Sequence of Geobacillus thermodenitrificans T12, A Potential Host for Biotechnological Applications
Source: Curr Microbiol. 2017 Sep 12;75(1):49–56. doi: 10.1007/s00284-017-1349-0 (PMC5765199; doi:10.1007/s00284-017-1349-0)
Supplement: Supplementary file 4 — Supplementary material 4 (DOCX 17 kb) [file 284_2017_1349_MOESM4_ESM.docx]

Table S3 Additional CAZymes identified on the genome of *G. thermodenitrificans* T12

| **Locus tag** | **Gene name** | **Product size (aa)** | **Subcellular Localization** | **Predicted protein function** | **Closest non-*Geobacillus* ortholog** | **Strain** | **AA identity (%)** | **Bitscore** | **E-value** |
| --- | --- | --- | --- | --- | --- | --- | --- | --- | --- |
| GTHT12_00075 | *malL_1* | 556 | - | oligo 1,6-glucosidase | *Anoxybacillus geothermalis* | - | 92 | 2796 | 0.00E+00 |
| GTHT12_00158 | *-* | 589 | Extracellular | Alpha- amylase | *Thermus* sp. | YBJ-1 | 87 | 2842 | 0.00E+00 |
| GTHT12_00162 | *-* | 512 | Cytoplasm | Alpha- amylase | *Anoxybacillus amylolyticus* | MR3C | 92 | 2479 | 0.00E+00 |
| GTHT12_01209 | *malL_2* | 564 | - | oligo 1,6-glucosidase | *Anoxybacillus geothermalis* | - | 93 | 2819 | 0.00E+00 |
| GTHT12_01331 | *bglA* | 471 | - | Beta-glucosidase | *Anoxybacillus geothermalis* | - | 95 | 2401 | 0.00E+00 |
| GTHT12_01664 | *aga* | 730 | - | alpha-galactosidase | *Parageobacillus* genomosp. | 1 | 81 | 3267 | 0.00E+00 |
| GTHT12_01847 | *arbB* | 456 | - | 6-phospho-beta-glucosidase | *Anoxybacillus* sp. | SK3-4 | 95 | 2262 | 0.00E+00 |
| GTHT12_02317 | *pulA* | 727 | - | Pullulanase | Mixed culture bacterium | AmyA1 | 99 | 3873 | 0.00E+00 |
| GTHT12_02694 | *chbF* | 448 | - | 6-phospho-beta-glucosidase | *Anoxybacillus geothermalis* | - | 95 | 2262 | 0.00E+00 |
| GTHT12_02696 | *bglH* | 479 | - | 6-phospho-beta-glucosidase | *Parageobacillus* genomosp. | 1 | 88 | 2299 | 0.00E+00 |
| GTHT12_02766 | *-* | 566 | - | Sucrose phosphorylase | *Parageobacillus* genomosp. | 1 | 82 | 2535 | 0.00E+00 |
| GTHT12_02767 | *mngB* | 888 | - | Alpha-mannosidase | *Parageobacillus* genomosp. | 1 | 77 | 3795 | 0.00E+00 |
| GTHT12_03754 | *-* | 494 |  | Levanase | *Parageobacillus toebii* | - | 99 | 2610 | 0.00E+00 |
